# Supplementary material for: Content-rich biological network constructed by mining PubMed abstracts
Source: BMC Bioinformatics. 2004 Oct 8;5:147. doi: 10.1186/1471-2105-5-147 (PMC528731; doi:10.1186/1471-2105-5-147)
Supplement: Additional File 2 — The original results of the above study (non-essential files are deleted to keep the file size under the limit set by BMC bioinformatics). [file 1471-2105-5-147-S2.bz2 › chilibotAdditionalFile2/dip10/755ID8631671E18141/html/EXBB_EXBD.html]

 


 **EXBB** and **EXBD** 
  
Found 56 abstracts in PubMed, retrieved 10.  
 

 What does Google say? 
 PDF only 
| .edu only 

---

**Interactive relationship** (e.g. stimulation, inhibition, etc)

**Neutral relationship**- The aim of this review is to describe an in vivo assay of the interactions taking place in the Tol Pal or TonB  **ExbB**   **ExbD**  envelope complexes in the periplasm of Escherichia coli and between them and colicins or g3p protein of filamentous bacteriophages.  Ref: 12423784 Biochimie< MedlineTA>Biochimie,
- The recent observation that  **ExbB**  exists in 3.5 fold molar excess relative to the molarity of  **ExbD**  in E. coli suggests the possibility of two types of complexes, those containing both  **ExbB**  and  **ExbD**  and those containing only  **ExbB** .  Ref: 12193634 J Bacteriol, 2002
- Together, these results support an alternative model where all of the  **ExbB**  in the cell occurs in complex with all of the  **ExbD**  in the cell.  Ref: 12193634 J Bacteriol, 2002

**Non-interactive relationship** (e.g. studied together, co-existance, homology, etc.)

- **ExbB**  and  **ExbD** , despite being encoded from the same operon, were not equimolar, being present at 2463 522 and 741 105 copies respectively.  Ref: 11967085 Mol Microbiol, 2002
- TonB, the energy transducer, appears to go through a cycle during energy transduction, with the absence of both  **ExbB**  and  **ExbD**  creating blocks at two points i in the inability of TonB to respond to the cytoplasmic membrane proton motive force and ii in the conversion of TonB from a high affinity outer membrane association to a high affinity cytoplasmic membrane association.  Ref: 12193634 J Bacteriol, 2002
- Reverse transcription RT PCR, using RNA from organisms grown under iron replete and iron restricted conditions, revealed that tonB,  **exbB** ,  **exbD** , tbpB and tbpA are transcribed as a single unit with expression being up regulated in response to iron restriction.  Ref: 12742718 Vet Microbiol, 2003
- PCR approaches allowed the amplification, cloning and sequencing of A. suis tonB,  **exbB** ,  **exbD** , tbpB and tbpA homologues.  Ref: 12742718 Vet Microbiol, 2003
- and two cytoplasmic membrane proteins with less defined roles,  **ExbB**  and  **ExbD** .  Ref: 11967085 Mol Microbiol, 2002
- The Ton system consisting of the proteins TonB,  **ExbB**  and  **ExbD**  is required for colicin and microcin import.  Ref: 12423780 Biochimie< MedlineTA>Biochimie, 2002
- In this study, we report the per cell numbers of TonB,  **ExbB** ,  **ExbD**  and FepA for cells grown under iron replete and iron limited conditions.  Ref: 11967085 Mol Microbiol, 2002
- Specifically, both  **ExbB**  and  **ExbD**  were found to be required for TonB to conformationally respond to proton motive force.  Ref: 12193634 J Bacteriol, 2002
- The energy is transduced to FecA by the inner membrane complex, TonB,  **ExbB** , and  **ExbD** .  Ref: 12618451 J Bacteriol, 2003
- **ExbB**  and  **ExbD**  proteins are part of the TonB dependent energy transduction system and are encoded by the exb operon in Escherichia coli.  Ref: 12193634 J Bacteriol, 2002
- A complex of three membrane spanning proteins TonB,  **ExbB**  and  **ExbD**  couples the chemiosmotic potential of the cytoplasmic membrane with siderophore uptake across the outer membrane.  Ref: 12409204 Biochim Biophys Acta, 2002
- Both  **ExbB**  and  **ExbD**  were also required for association of TonB with the cytoplasmic membrane.  Ref: 12193634 J Bacteriol, 2002
- The ratio of these proteins was calculated at one TonB two  **ExbD**  seven  **ExbB**  under all four growth conditions tested.  Ref: 11967085 Mol Microbiol, 2002
- **ExbB**  and  **ExbD**  do not function independently in TonB dependent energy transduction.  Ref: 12193634 J Bacteriol, 2002
- Quantification of known components of the Escherichia coli TonB energy transduction system TonB,  **ExbB** ,  **ExbD**  and FepA.  Ref: 11967085 Mol Microbiol, 2002
- In gram negative organisms, high affinity transport of iron substrates requires energy transduction to specific outer membrane receptors by the TonB  **ExbB**   **ExbD**  complex.  Ref: 12562789 J Bacteriol, 2003
- Pasteurella multocida  **exbB** ,  **exbD**  and tonB genes are physically linked but independently transcribed.  Ref: 12044675 FEMS Microbiol Lett, 2002
- The  **exbB** ,  **exbD**  and tonB genes of the Pasteurella multocida animal pathogen have been cloned by complementation of an Escherichia coli tonB mutant.  Ref: 12044675 FEMS Microbiol Lett, 2002
- Crystal structures of two outer membrane transport proteins, FhuA and FepA, and biochemical data reveal strong long range conformational changes of the proteins upon binding of Fe 3 siderophore complexes and in response to energy transfer from the cytoplasmic membrane into the outer membrane via the TonB  **ExbB**   **ExbD**  protein complex.  Ref: 11934617 Curr Opin Microbiol, 2002
